# Brain Ultrasonography Findings in Neonatal Seizure; a Cross-sectional Study

**Published:** 2017-01-12

**Authors:** Seyed Saeed Nabavi, Parinaz Partovi

**Affiliations:** 1 Clinical Research Development Center, Amir-Almomenin Hospital, Islamic Azad University, Tehran Medical Sciences Branch, Tehran, Iran.

**Keywords:** Seizures, infant, newborn, ultrasonography, diagnosis, brain

## Abstract

**Introduction:**

Screening of newborns with seizure, who have curable pathologic brain findings, might be able to improve their final outcome by accelerating treatment intervention. The present study aimed to evaluate the brain ultrasonography findings of newborns hospitalized with complaint of seizure.

**Methods:**

The present cross-sectional study designed to evaluate brain ultrasonography findings of hospitalized newborns complaining seizure. Neonatal seizure was defined as presence of tonic, clonic, myoclonic, and subtle attacks in 1 - 28 day old newborns.

**Results:**

100 newborns with the mean age of 5.82 ± 6.29 days were evaluated (58% male). Most newborns were in the < 10 days age range (76%), term (83%) and with normal birth weight (81%). 22 (22%) of the ultrasonography examinations showed a pathologic finding. A correlation was only found between birth age and probability of the presence of a pathologic problem in the brain as the frequency of these problems was significantly higher in pre-term newborns (p = 0.023).

**Conclusion:**

Based on the findings of the present study, frequency of pathologic findings in neonatal brain ultrasonography was 22%. Hemorrhage (12%) and hydrocephaly (7%) were the most common findings. The only factor correlating with increased probability of positive findings was the newborns being pre-term.

## Introduction

Seizure is the most common neurologic problem in infants, recurrence of which can cause disturbance in the central nervous system growth process. Most cases of seizure are idiopathic; but neonatal seizure is usually a sign of a pathologic problem in the brain that may be accompanied by a permanent damage in future stages of life (1, 2). Prevalence of newborn seizure is reported to be between 1.8 and 8.6 in each 1000 live births (2). The considerable difference in the reported statistics can be related to problem in diagnosis, different definitions of neonatal seizure, and various populations. Sometimes determining which clinical phenomenon should be considered to be seizure is a difficult task (3). The most common causes of neonatal seizure are hypoxia-ischemia (asphyxia), brain stroke, intra-ventricular or intracranial hemorrhage, meningitis, sepsis, and metabolic disorders (4, 5). Recent studies have shown that the nervous system of newborns may be resistant to long-lasting seizures to some extent; however, recurrent short seizures may be associated with permanent damage to the central nervous system, increased risk of epilepsy and durable cognitive disabilities (1, 6). Screening of newborns with seizure, who have curable pathologic brain findings, might be able to improve their final outcome by accelerating treatment intervention. Electroencephalogram (EEG) has been reported to be abnormal in 100% of clonic, partial tonic, and spasmic seizures; 60% of generalized myoclonic ones; 7% of focal and multifocal myoclonic, and 10% of generalized tonic seizures. Meanwhile, silent, apnostic, and autonomic types of seizure do not have a known correlation with EEG findings (7). Brain magnetic resonance imaging (MRI), computed tomography (CT) scan, and EEG, despite having a high accuracy are not available everywhere and require special conditions such as immobilization. Currently, ultrasonography is considered to be used for various purposes such as measuring intracranial pressure (8, 9), fracture diagnosis (10-12), etc. in emergency setting (13, 14). Although the ability of ultrasonography in detection of newborn intracranial lesions has been introduced during the 1990s, it has not been seriously considered until now, especially in third world countries (15). It seems that ultrasonography as a safe, affordable, available and bedside screening tool can be of great help for physicians in charge of such patients (16-19). Therefore, the present study aimed to evaluate the brain ultrasonography findings of newborns hospitalized with complaint of seizure.

## Methods

***Study design and setting***

The present study is a retrospective cross-sectional one aiming to evaluate brain ultrasonography findings of newborns hospitalized in the neonatal unit of Milad Hospital, Tehran, Iran, during 2011 to 2013 complaining seizure. Researchers adhered to principles of Helsinki declaration and confidentiality of patient data. This study was approved by the ethics committee of Islamic Azad University, Tehran Medical Sciences branch.

***Participants***

Newborns hospitalized in neonatal unit following seizure were studied using convenience sampling. Age between 1 and 28 days; presence of tonic, clonic, myoclonic, and autonomic; no missing data in the clinical profile of the newborn; availability of data on brain ultrasonography of the newborn, and not having history of trauma were among the inclusion criteria. Newborns with a history of apnea due to pulmonary, cardiac, digestive, and infectious problems as well as those who did not have a definite diagnosis of seizure were excluded from the study.

***Data gathering***

Using the clinical profile of the patients, a checklist consisting of demographic data including age, sex, birth weight, birth age, family history of neonatal seizure, history of underlying illnesses, and type of birth as well as brain ultrasonography findings was filled for each of them. Neonatal seizure was defined as presence of tonic, clonic, myoclonic, and subtle attacks based on Mizrahi and Kellawaycriteria in 1 - 28 day old patients (20). Data gathering was done by a trained medicine student.

Ultrasonography was done using a 3.5 – 5 MHz curve probe. All ultrasonography examinations were performed from the anterior fontanelle, by a single radiologist. 

***Statistical analysis***

Data analysis was done using SPSS version 13. Quantitative data were reported as mean and standard deviation (SD) and qualitative ones as frequency and percentage. For evaluating the correlation between ultrasonography findings and demographic data of the patients, chi-square and ANOVA tests were used. P< 0.05 was considered as significance level.

## Results

100 newborns with the mean age of 5.82 ± 6.29 days were evaluated (58% male). Table 1 depicts the baseline characteristics of the studies patients. Most newborns were in the < 10 days age range (78%), term (83%) and with normal birth weight (81%). 6 (6%) newborns had a history of seizure and 27 (27%) cases had an underlying illness. Table 2 shows the brain ultrasonography findings of the studied newborns. 22 (22%) of the ultrasonography examinations showed a pathologic finding. Table 3 demonstrates the correlation between ultrasonography findings and demographic data of the patients. A correlation was only found between birth age and probability of the presence of a pathologic problem in the brain as the frequency of these problems was significantly higher in pre-term newborns (p = 0.023).

## Discussion

Based on the findings of the present study, 22% of the hospitalized newborns in the neonatal unit of the studied hospital had at least 1 pathologic finding in their brain ultrasonography. Hemorrhage and hydrocephaly with 12 and 7 cases were the most common ultrasonography findings, respectively. The only factor correlating with increased probability of positive findings in brain ultrasonography was the newborns being pre-term.

**Table 1:** Baseline characteristics of the studied newborns

Variable	**Number (%)**
**Age (days)**	
0 – 9.9	78 (76)
10 – 19.9	13 (13)
20 – 28	9 (9)
**Birth weight (gr)**	
Low (< 2500)	16 (16)
Normal (2500 – 4000)	81 (81)
High (> 4000)	3 (3)
**Delivery type**	
Natural	33 (33)
Cesarean section	67 (67)
**Birth age**	
Term	83 (83)
Pre-term	17 (17)
**Family history of seizure**	
Yes	6 (6)
No	94 (94)
**Underlying problem**	
Yes	27 (27)
No	73 (73)

**Table 2:** Brain ultrasonography findings of the newborns with seizure

Variable	**Number (%)**
Normal	78 (78)
Hemorrhage	12 (12)
Hydrocephaly	7 (7)
Other	3 (3)

**Table 3:** The correlation between ultrasonography findings and baseline characteristics of the newborns with seizure

**variable**	Pathologic findings n (%)		**P value**
	**Yes**	**No**	
**Sex **			
Male	14 (24.1)	44 (75.9)	0.544
Female	8 (19)	34 (81)	
**Birth weight (gr)**			
Low (< 2500)	5 (31.2)	11 (68.8)	0.343
Normal (2500 – 4000)	16 (19.8)	65 (80.2)	
High (> 4000)	1 (23.3)	2 (67.7)	
**Delivery type**			
Natural	7 (21.2)	26 (78.8)	0.894
Cesarean section	15 (22.4)	52 (77.6)	
**Birth age**			
Term	22 (10.1)	68 (81.9)	0.023
Pre-term	7 (41.2)	10 (58.8)	

In a study by Zahid et al. carried out in 2010 and 2011 for evaluation of brain ultrasonography findings in newborns with seizure, 48.5% of the performed ultrasonography examinations had pathologic findings such as: intraventricular hemorrhage (27.6%), brain edema (11.7%), subdural hemorrhage (6.4%), and subarchanoid hemorrhage (5.3%). They introduced ultrasonography as a proper non-invasive method for timely diagnosis of cerebral causes of seizure in newborns (19). In our study, although the number of ultrasonography examinations with positive finding was half the rate reported in the mentioned study, 22% prevalence of ultrasonography findings was also important. In Leth et al. study, based on brain ultrasonography findings, the cause of 10% of newborn seizures was determined to be brain lesions, while this rate rose to 68% after performing MRI. In the study, 35% of the seizures were reported to be due to hypoxic problems, 26% hemorrhagic, 16% metabolic disorders and unknown in 23% (16). In a study by Mercuri et al. 11 out of 16 newborns with seizure (69%) had pathologic lesions in their brain ultrasonography, which were mostly hemorrhagic in the initial weeks and ischemic after that (17). This rate was estimated to be 95% in Rutherford et al. study. Small infarcts that had not been detected in ultrasonography were seen in MRI (18).

Wang et al. in their study in 2004 concluded that ultrasonographic screening of brain for all newborns can be helpful in detection of rare but important problems affecting neurologic outcomes (21).

Considering the vastly different results that exist regarding the rate of pathologic findings detected by brain ultrasonography, it seems that further studies with larger sample sizes and more accurate methodologies are required before making a final decision in this regard. In addition, to determine the screening performance characteristics of this test, it should be compared to more standard diagnostic tests such as MRI and determine its sensitivity, specificity, and accuracy.

## Conclusion:

Based on the findings of the present study, frequency of pathologic findings in neonatal brain ultrasonography was 22%. Hemorrhage (12%) and hydrocephaly (7%) were the most common findings. The only factor correlating with increased probability of positive findings was the newborns being pre-term.
